# Push-pull strategies for vector control

**DOI:** 10.1186/1475-2875-9-S2-I16

**Published:** 2010-10-20

**Authors:** Willem Takken

**Affiliations:** 1 Laboratory of Entomology, Wageningen University, 6700 EH Wageningen, The Netherlands

## 

In many disease vectors, olfaction is the principal modality with which food sources (plants and vertebrates) and oviposition sites are being identified [1]. Chemical cues emanating from these sources are detected from a distance, often inducing a behavioural response that leads the arthropod vector to the source. At close range, other sensual modalities such as vision, heat and moisture may affect this behaviour as well. Arthropods can identify these chemical cues from a long distance, in some cases over 100m or further. The most common odorant cue is carbon dioxide (CO_2_), which is recognised by most blood feeding arthropods. Other chemical odorants are more group- or even species-specific, for example tsetse flies respond to CO_2_ and blend of 1-octen-3-ol, 4-Methylphenol and 3-n-Propylphenol [2], malaria mosquitoes to ammonia, lactic acid and tetradecanoic acid [3] and yellow fever mosquitoes to ammonia, lactic acid and caproic acid [4]. Pathogens of human and animal disease make use of these odorants by hitchhiking with the vectors, to be passed on from one host to the next. The vertebrate hosts, therefore, produce chemicals that favour the transmission of infectious disease.

While many animals produce odorants that are perceived as attractive by the arthropods, some odorants cause repellent effects, pushing the arthropods away from the source and thus protecting the animals against insect bites and potential disease transmission. Recent advances in chemical ecology have led to the development of technologies where pest insects, including disease vectors, can be manipulated by a skilful combination of attractants and repellents that lead the vectors to traps or killing devices. This "push-pull" strategy [5] has already caused significant reductions of transmission of trypanosomiasis, and it is considered as a control method targeting other vector-borne disease complexes. Exploitation of behavioural characteristics of the vectors hence leads to reduced disease transmission and, in some cases, even disease elimination. The presentation will discuss the state-of-the-art of this behavioural technology for vector-borne disease control, and provide an outlook for future prospects.
